# DarkHorse: a method for genome-wide prediction of horizontal gene transfer

**DOI:** 10.1186/gb-2007-8-2-r16

**Published:** 2007-02-02

**Authors:** Sheila Podell, Terry Gaasterland

**Affiliations:** 1 Scripps Genome Center, Scripps Institution of Oceanography, University of California at San Diego, Gilman Drive, La Jolla, CA 92093-0202, USA

## Abstract

A new approach to rapid, genome-wide identification and ranking of horizontal transfer candidate proteins is presented. The method is quantitative, reproducible, and computationally undemanding. It can be combined with genomic signature and/or phylogenetic tree-building procedures to improve accuracy and efficiency. The method is also useful for retrospective assessments of horizontal transfer prediction reliability, recognizing orthologous sequences that may have been previously overlooked or unavailable. These features are demonstrated in bacterial, archaeal, and eukaryotic examples.

## Abstract 2

DarkHorse is a new approach to rapid, genome-wide identification and ranking of horizontal transfer candidate proteins.

## Background

Horizontal gene transfer can be defined as the movement of genetic material between phylogenetically unrelated organisms by mechanisms other than parent to progeny inheritance. Any biological advantage provided to the recipient organism by the transferred DNA creates selective pressure for its retention in the host genome. A number of recent reviews describe several well-established pathways of horizontal transfer [1-4]. Evidence for the unexpectedly high frequency of horizontal transmission has spawned a major re-evaluation in scientific thinking about how taxonomic relationships should be modeled [4-9]. It is now considered a major factor in the process of environmental adaptation, for both individual species and entire microbial populations. Horizontal transfer has also been proposed to play a role in the emergence of novel human diseases, as well as determining their virulence [10,11].

There is currently no single bioinformatics tool capable of systematically identifying all laterally acquired genes in an entire genome. Available methods for identifying horizontal transfer generally rely on finding anomalies in either nucleotide composition or phylogenetic relationships with orthologous proteins. Nucleotide content and phylogenetic relatedness methods have the advantage of being independent of each other, but often give completely different results. There is no 'gold standard' to determine which, if either, is correct, but it has been suggested that different methodologies may be detecting lateral transfer events of different relative ages [2,12].

In addition to having good sensitivity and specificity, ideal tools for identifying horizontal transfer at the genomic level should be computationally efficient and automated. The current environment of rapid database expansion may require analyses to be re-performed frequently, in order to take advantage of both new genome sequences and new annotation information describing previously unknown protein functions. Re-analysis using updated data may provide new insights, or even change conclusions completely.

A variety of strategies have been used to predict horizontal gene transfer using nucleotide composition of coding sequences. Early methods flagged genes with atypical G + C content; later methods evaluate codon usage patterns as predictors of horizontal transfer [13-15]. A variety of so called 'genomic signature' models have been proposed, using nucleotide patterns of varying lengths and codon position. These models have been analyzed both individually and in various combinations, using sliding windows, Bayesian classifiers, Markov models, and support vector machines [16-19].

One limitation of nucleotide signature methods is that they can suggest that a particular gene is atypical, but provide no information as to where it might have originated. To discover this information, and to verify the validity of positive candidates, signature-based methods rely on subsequent validation by phylogenetic methods. These cross-checks have revealed many clear examples of both false positive and false negative predictions in the literature [20-23].

The fundamental source of error in predictions based on genomic signature methods is the assumption that a single, unique pattern can be applied to an organism's entire genome [24]. This assumption fails in cases where individual proteins require specialized, atypical amino acid sequences to support their biological function, causing their nucleotide composition to deviate substantially from the 'average' consensus for a particular organism. Ribosomal proteins, a well known example of this situation, must often be manually removed from lists of horizontal transfer candidates generated by nucleotide-based identification methods [25].

The assumption of genomic uniformity is also incorrect in the case of eukaryotes that have historically acquired a large number of sequences through horizontal transfer from an internal symbiont, or an organelle like mitochondrion or chloroplast. For example, the number of genes believed to have migrated from chloroplast to nucleus represents a substantial portion of the typical plant genome [26]. In this case, patterns of nucleotide composition should fall into at least two distinct classes, requiring multiple training sets to build successful models using machine learning algorithms. To avoid this complexity, many authors propose limiting application of their genomic signature methods to simple prokaryotic or archaeal systems.

Phylogenetic methods seek to identify horizontal transfer candidates by comparison to a baseline phylogenetic tree (or set of trees) for the host organism. Baseline trees are usually constructed using ribosomal RNA and/or a set of well-conserved, well-characterized protein sequences [27]. Each potential horizontal transfer candidate protein is then evaluated by building a new phylogenetic tree, based on its individual sequence, and comparing this tree to the overall baseline for the organism. Unexpectedness is usually defined as finding one or more nearest neighbors for the test sequence in disagreement with the baseline tree. More recently, a number of automated tree building methods have used statistical approaches to identify trees for individual genes that do not fit a consensus tree profile [28-32].

Although phylogenetic trees are generally considered the best available technique for determining the occurrence and direction of horizontal transfer, they have a number of known limitations. Analysts must choose appropriate algorithms, out-groups, and computational parameters to adjust for variability in evolutionary distance and mutation rates for individual data sets. Results may be inconclusive unless a sufficient number and diversity of orthologous sequences are available for the test sequence. In some cases, a single set of input data may support multiple different tree topologies, with no one solution clearly superior to the others. Building trees is especially challenging in cases where the component sequences are derived from organisms at widely varying evolutionary distances.

Perhaps the biggest drawback to using tree-based methods for identifying horizontal transfer candidates is that these methods are very computationally expensive and time consuming; it is currently impractical to perform them on large numbers of genomes, or to update results frequently as new information is added to underlying sequence databases. Even a relatively small prokaryotic genome requires building and analyzing thousands of individual phylogenetic trees. To manage this computational complexity, many authors exploring horizontal transfer events have been forced to limit their calculations to one or a few candidate sequences at a time.

More recently, semi-automated methods have become available for building multiple phylogenetic trees at once [33,34]. These methods are suitable for application to whole genomes, and include screening routines to identify trees containing potential horizontal transfer candidates. However, to achieve reasonable sensitivity without an unacceptable false positive rate, these methods still require each candidate tree identified by the automated screening process to be manually evaluated. One recent publication described the automated creation of 3,723 trees, of which 1,384 were identified as containing potential horizontal candidates [35]. After all 1,384 candidate trees were inspected manually, approximately half were judged too poorly resolved to be useful in making a determination. Of the remaining trees, only 31 were ultimately selected as containing horizontally transferred proteins. Despite the Herculean effort involved in producing these data, the authors concluded that it was only a 'first look' at horizontal transfer, which would need to be repeated when more sequence data became available for closely related organisms.

Given the time and difficulty of creating phylogenetic trees from scratch, a tool that automatically coupled amino acid sequence data with known lineage information could avoid an enormous amount of repetitive effort in re-calculating well-established facts. It is, therefore, somewhat surprising that currently available methods do not generally take advantage of resources like the NCBI Taxonomy database, which links phylogenetic information for thousands of different species to millions of protein sequences. One notable exception has been the work of Koonin *et al*. [1], who searched for horizontal transfer in 31 bacterial and archaeal genomes by a combination of BLAST searches with semi-automated and manual screening techniques. To avoid false positive results, these authors felt it necessary to manually check every 'paradoxical' best hit, in many cases amounting to several hundred matches per microbial genome. While this strategy undoubtedly improved the quality of results presented, the extensive amount of time and labor required for manual inspection precludes applying the techniques used by these authors to larger eukaryotic genomes, or to the hundreds of new microbial genomes sequenced since 2001.

One potential problem in using taxonomy database information as a horizontal transfer identification tool is the difficulty of establishing reliable surrogate criteria for orthology, which might avoid the need for extensive re-building of phylogenetic trees. It is well known that 'top hit' sequence alignments identified by the BLAST search algorithm do not necessarily return the phylogenetically most appropriate match [36]. In addition to incorrect ranking of BLAST matches, other difficulties to be overcome include differences in BLAST score significance due to mutation rate variability, unequal representation of different taxa in source databases, and potential gene loss from closely related species [37]. Finally, any detection system dependent on identifying phylogenetically distant matches may sacrifice sensitivity in detecting horizontal transfer between closely related organisms.

To address these issues, the DarkHorse algorithm combines a probability-based, lineage-weighted selection method with a novel filtering approach that is both configurable for phylogenetic granularity, and adjustable for wide variations in protein sequence conservation and external database representation. It provides a rapid, systematic, computationally efficient solution for predicting the likelihood of horizontally transferred genes on a genome-wide basis. Results can be used to characterize an organism's historical profile of horizontal transfer activity, density of database coverage for related species, and individual proteins least likely to have been vertically inherited. The method is applicable to genomes with non-uniform compositional properties, which would otherwise be intractable to genomic signature analysis. Because the procedure is both rapid and automated, it can be performed as often as necessary to update existing analyses. Thus, it is particularly useful as a screening tool for analyzing draft genome sequences, as well as for application to organisms where the number of database sequences available for taxonomic relatives is changing rapidly. Promising results can be then prioritized and analyzed in more depth using independent criteria, such as nucleotide composition, manual construction of phylogenetic trees, synteneic neighbor analysis, or other more detailed, labor-intensive methods.

## Results

### Algorithm overview

**Figure 1:**
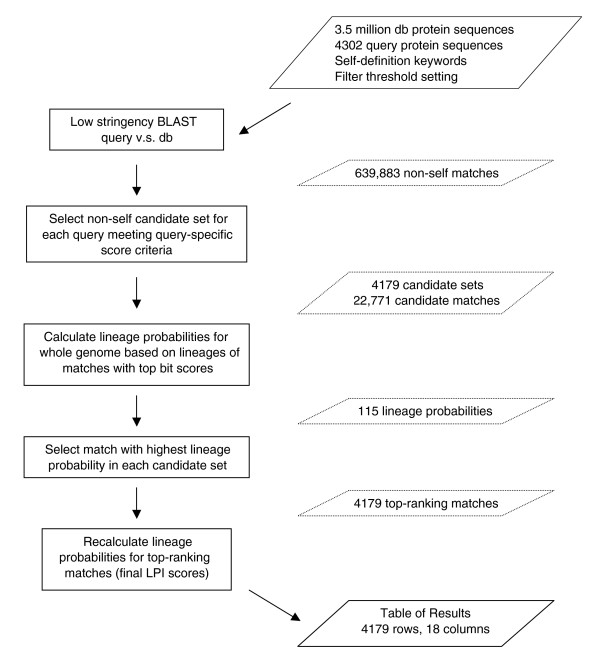
Flow diagram illustrating DarkHorse work flow, with example numbers for *Escherichia coli *strain K12. Parallelograms indicate data, rectangles indicate processes. Parallelograms with dashed borders indicate intermediate data, output by one step and input to the next step.

Figure 1 illustrates the basic steps in analyzing a genome using the DarkHorse algorithm, with *Escherichia coli *strain K12 as an example. In addition to protein sequences from the test genome and a reference database, program input includes two user-modifiable parameters: a list of self-definition keywords and/or taxonomy id numbers, and a filter threshold setting. The self-definition keywords determine phylogenetic granularity of the search and relative age of potential horizontal transfer events being examined. The filter threshold setting is a numerical value used to adjust stringency for relative database abundance or scarcity of sequences from species closely related to the test genome. These parameters can be varied independently or iteratively in repeated runs to fine-tune the scope of the analysis.

The process begins with a low stringency BLAST search, performed for all predicted genomic proteins against the reference database. All BLAST matches containing self-definition keywords and/or taxonomy id numbers are eliminated from these search results. For each genomic protein, the remaining BLAST alignments are filtered to select a candidate match set, based on both query-specific BLAST scores and the global filter threshold setting. Database proteins with the maximum bit score from each candidate set are used to calculate preliminary 'lineage probability index' (LPI) scores. LPI is a new metric introduced in this paper that is key to the genome-wide identification of horizontally transferred candidates. Organisms closely related to the query genome receive higher LPI scores than more distant ones, and groups of phylogenetically related organisms receive similar scores to each other, regardless of their abundance or scarcity in the reference database. Details of the procedure used to calculate LPI scores are presented in the Materials and methods section.

Preliminary LPI scores are used to re-order the candidate sets, now choosing the candidate with the maximum LPI score from each set as top-ranking. These revised top-ranking matches are then used to refine preliminary LPI scores in a second round of calculation. Final results are presented in a tab-delimited table of results. An example of the program's tab-delimited output is provided as Additional data file 1.

GenBank nr was chosen as the reference database for this study to obtain the widest possible diversity of potential matches, but the algorithm could alternatively be implemented using narrower or more highly curated databases. The set of query protein sequences must be large enough to fairly represent the full range of diversity present in the entire genome. The easiest way to ensure unbiased sampling is to include all predicted protein sequences from a genome, but this requirement might also be met in other ways, for example, with a large set of cDNA sequences. Blast searches performed using predicted amino acid sequences were found to be more useful than nucleic acid searches, resulting in fewer false positive matches and giving a more favorable signal/noise ratio.

Parameter settings for the preliminary BLAST search are used as a coarse filter to reduce computation time and memory requirements, removing low scoring matches as early as possible. These initial settings need to be broad enough to include even very distant orthologs, but do not affect final LPI scores as long as no true protein orthologs have been prematurely eliminated. To reduce the frequency of single-domain matches to multi-domain proteins, initial filtering for this study included a requirement for each match to cover at least 60% of the query sequence length. BLAST bit score was used as a metric for subsequent ranking and filtering steps, to ensure fairness in analyzing sequences of varying lengths.

### Selection and ranking of candidate match sets

One well-known problem in using the BLAST search algorithm to rank candidate matches is that highly conserved proteins can generate multiple database hits with similar scores, and quantitative differences between the first hit and many subsequent matches may be statistically insignificant. No single, absolute threshold value is suitable as a significance cutoff for all proteins within a genome, because degree of sequence conservation varies tremendously. In addition to variability among proteins, mutation rates and database representation can also vary widely between taxa, so appropriate threshold values may need adjustment by query organism, as well as by individual protein.

To overcome these problems, DarkHorse considers bit score differences relative to other BLAST matches against the same genomic query, rather than considering absolute differences. For each query protein, a set of ortholog candidates is generated by selecting all matches that fall within an individually calculated bit score range. The minimum of this range is set as a percentage of the best available score for any non-self hit against that particular query. The percentage is equal to the global filter threshold setting chosen by the user, which can, in theory, vary between 0% and 100%. A zero value requires that all candidate matches for a particular query have bit scores exactly equal to the top non-self match. Filter threshold settings intermediate between 0% and 100% require that candidate matches have bit scores in a range within the specified percentage of the highest scoring non-self match. In practice, values between 0% and 20% are found to be most useful in identifying valid horizontal transfer candidates. The effects of threshold settings on the phylogeny of top-ranking candidates are illustrated for genomes from four different organisms in Tables 1 to 7.

Once candidate match sets have been selected for each genomic protein, lineage information is retrieved from the taxonomy database. This information is used to calculate preliminary estimates of lineage frequencies among potential database orthologs of the query genome. These preliminary estimates are used as guide probabilities in a first round of candidate ranking, then later refined in a second round of ranking.

The probability calculation procedure, described in detail in the Materials and methods section, is based on the average relative position and frequency of lineage terms. More weight is given to broader, more general terms occurring at the beginning of a lineage (for example, kingdom, phylum, class), and less weight to narrower, more detailed terms that occur at the end (for example, family, genus, species). To compensate for the fact that some lineages contain more intermediate terms than others (for example, including super- and/or subclasses, orders, or families), the calculation normalizes for total number of terms, and weights each term according to its average position among all lineages tested, rather than an absolute taxonometric rank. The end result is a very fast, computationally simple technique to assign higher probability scores to lineages that occur more frequently, and lower scores to lineages that occur only rarely. Groups of phylogenetically related organisms receive similar lineage probability scores, even if actual matches to the query genome are unevenly distributed among individual members of the group.

The probability calculation is performed twice during each search for horizontal transfer candidates, once to obtain a set of preliminary guide probabilities, and a second time to obtain more refined LPI scores. Initial guide probabilities are calculated using one sequence from each candidate match set, selected on the basis of having the highest BLAST bit score in the set. Once guide probabilities are established, they are used to re-rank the members of each candidate set by lineage probability instead of bit score, in some cases resulting in the choice of a new top-ranking sequence. The lineage-probability calculation is then repeated using the revised set of top-ranking candidates as input, to obtain final LPI scores, which range between zero and one. Additional rounds of probability calculation and candidate selection would be possible but are unnecessary; lineage probability scores generally change only slightly between the preliminary guide step and final LPI assignments.

### Filter threshold optimization

Selecting a global filter threshold value of zero maximizes the opportunity to identify horizontal transfer candidates, but may result in false positives if sequences from closely related organisms have BLAST scores that are slightly, but not significantly, lower than the top hit. Using a higher value for the threshold filter, allowing a wider range of hits to be considered in the candidate set for each query, helps eliminate false positive horizontal transfer candidates by promoting matches from closely related species over those from more distant species. However, as the range of acceptable scores for match candidates is progressively broadened, sensitivity to potential horizontal transfer events is correspondingly decreased, and true examples of horizontal transfer may be overlooked.

The effects of filter threshold cutoff settings on phylogenetic distribution of corrected best matches were examined in detail for *E. coli *strain K12. In this example, all protein matches to the genus *Escherichia *were excluded under the user-specified definition of self. In addition, matches containing the terms 'cloning', 'expression', 'plasmid', 'synthetic', 'vector', and 'construct' were also excluded to remove artificial sequences that might originally have been derived from *E. coli*.

Table 1 summarizes the *E. coli *filter threshold results. BLAST matches above the initial screening threshold were found for 4,179 (97%) of the original 4,302 genomic query sequences. With a filter threshold cutoff of 0%, the great majority of lineage-corrected best matches are closely related Enterobacterial proteins, as expected. As the filter threshold is progressively broadened, this number increases from 4,000 to a maximum of 4,112, reflecting the promotion of matches from closely related species to a best candidate position. However, some *E. coli *proteins had no matches to Enterobacterial database entries, even at a filter threshold setting of 100%, where all BLAST hits above the initial screening minimum are considered equivalent. Matches to these sequences are found only in phage, eukaryotes, and more distantly related bacteria, and represent either database errors, gene loss in all other sequenced members of this lineage, hyper-mutated sequences unique to this strain of *E. coli*, or candidates for lateral acquisition.

Table 2 shows detailed information for the eight eukaryotic sequences initially identified as best matches to *E. coli*. For each *E. coli *query sequence, the top hit match using a 0% threshold is shown first (bold). The second line for the same query (italicized) shows results at the lowest filter value where an alternative match with a higher LPI score was found. In five cases, increasing the filter threshold revealed additional BLAST matches to sequences with higher LPI values, suggesting the original match might be incorrect. In three cases, no better match was found, supporting statistical validity of the original result.

Interpreting BLAST search results for *E. coli *requires caution, because there is an especially high risk of finding matches to contaminating cloning vector and host sequences in genomic data for other organisms. This problem is illustrated by the first entry in Table 2, for the *E. coli *beta-galactosidase protein AAC74689, a common cloning vector component. The top ranking match for this query at a filter value of zero is *Arabidopsis *protein CAC43289. The BLAST alignment for this match is excellent, with 99% identity over all 603 amino acids of the query sequence, but application of a filter threshold setting of 2% reveals another extremely good match in the database, ZP_00698534 from *E. coli*'s close relative *Shigella boydii*. In the original BLAST analysis, the *Shigella *protein received a bit score of 1,255, compared to 1,261 for the *Arabidopsis *protein, even though both proteins have the same percent identity and query coverage length. Clearly this difference in bit score is insignificant, and difficult to detect without adequate surveillance. Ranking the matches by decreasing LPI score solves this problem; the *Arabidopsis *match has an LPI score of 0.009, but the *Shigella *match has an LPI score of 0.98. This example shows how a combination of threshold range filtering and LPI score ranking can successfully eliminate false positive artifacts due to cloning vector contamination.

The second and third queries in Table 2, for the enzymes mannitol phosphate dehydrogenase and cytosine deaminase, also appear to have matched inappropriate database sequences when using a zero threshold setting. Using a filter threshold of 20% or lower overcomes these apparent errors, replacing them with nearly equal matches in a species closely related to the original query organism. In contrast, the fifth query of Table 2 (AAC75891) illustrates the danger of setting threshold values that are too lenient. In this case, using a filter threshold of 80%, a BLAST hit from a phylogenetically closer organism (*Salmonella*) has been promoted even though it has only 28% identity to the query, versus 85% in the original top hit. This promotion is clearly unjustified.

**Figure 2:**
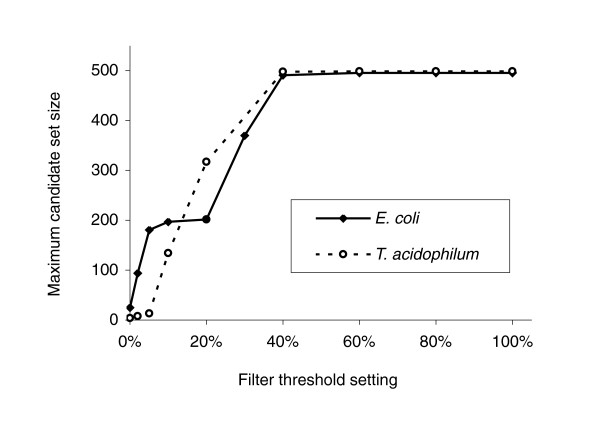
Effect of filter threshold setting on maximum number of candidate set members per query.

For optimal DarkHorse performance, threshold values need to be set at a level that is neither too high nor too low. The best threshold setting for an individual query organism depends on the abundance of closely related sequences in the database used for BLAST searches. This value is difficult to measure directly, but can be calibrated approximately by measuring the maximum candidate set size returned using different threshold settings on a genome-wide basis, as shown in Figure 2. For this data set, the original BLAST search included a maximum possible number of 500 matches per query. Values shown in the graph indicate the highest number of candidate matches found for any single query in the test genome after filtering at the indicated threshold setting.

For an organism like *E. coli*, with sequences available for many closely related species, the maximum number of candidate set members appears to reach a plateau when using a filter threshold setting of 10% to 20%. After that point, further broadening of the threshold compromises the effectiveness of the filtering process. For query organisms from more sparsely represented phylogenetic groups, such as the archaeon *Thermoplasma acidophilum*, there are very few examples of closely related species in the database. In these cases, a lower filter threshold cutoff value is appropriate. For some organisms, it may make sense to limit the filter threshold setting to zero, promoting only those matches whose scores are exactly equivalent to the initial top hit.

Threshold filtering can help eliminate statistical anomalies of BLAST scoring, but there are some types of database ambiguities it cannot resolve. One such example is the sixth entry in Table 2, a match between *E. coli *sequence AAC73796 and database entry BAB33410, isolated from snow pea pods (*P. sativum*). This match covers 100% of the *E. coli *query sequence at 100% identity, but only 46% of the pea protein. Sequences distantly related to the matched region exist in several other strains of *E. coli *and *Shigella*, but were not recognized by threshold filtering because they fall below the minimum BLAST match retention criteria. No related sequences are found in any eukaryotes other than snow pea, even at an e-value of 10.0. If this were a true case of horizontal transfer, closeness of the match would imply a very recent event, and phylogenetic distribution would suggest direction of transfer as moving from *E. coli *to the seed pods of a eukaryotic plant. But this scenario is biologically unlikely. A more reasonable explanation is that the sequence identity is due to an undetected artifact introduced during cloning of the pea sequence. This sequence was obtained from a single isolated cDNA clone, and reported in a lone, unverified literature reference [38]. This type of error is difficult to avoid in uncurated databases like GenBank nr.

### Definition of database 'self' sequences

The definition of 'self' sequences for a query organism is configured by a list of user-defined self-exclusion terms. These terms, which can be either names or taxonomy ID numbers, provide a simple way to adjust phylogenetic granularity of the search, and to compensate for over-representation of closely related sequences in the source database. Although the LPI scoring method is naturally more sensitive to transfer events between distantly related taxa than to closely related species, adjusting breadth of the self-definition keywords for a test organism can reveal potential horizontal transfer events that are either very recent or progressively more distant in time. In practice, this is accomplished by choosing a narrow initial self-definition, then iteratively adding one or more species with high LPI scores to the list of self-definition keywords in the next round of analysis. Query sequences acquired since the divergence of two related genomes can be identified by comparing LPI scores and associated lineages plus or minus one of the relatives as a self-exclusion term.

**Figure 3:**
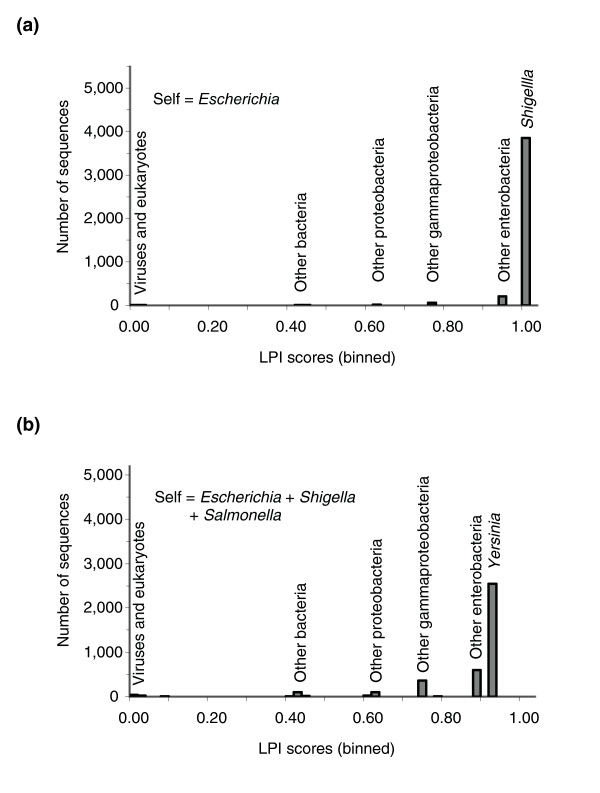
Effect of expanding *E. coli *self definition terms on LPI score distribution histograms. Filter threshold setting was 10%. (a) Self = *Escherichia *(b) Self = *Escherichia *+ *Shigella *+ *Salmonella*.

As an example of this process, the self definition for *E. coli *strain K12 was first defined narrowly by a set of strain-specific names and NCBI taxonomy ID numbers (K12, 83333, 316407, 562). This self-definition includes strain K12, as well as matches where the *E. coli *strain is unspecified, but still permits matches to clearly identified genomic sequences from alternative strains, for example, O157:H7. A second self-definition list was created using genus name *Escherichia *alone, which eliminates all species and strains from this genus. The list was then iteratively broadened by adding the names *Shigella *and *Salmonella*. Table 3 illustrates how this process changes the lineages of best matches chosen by DarkHorse. As the breadth of self-definition terms is expanded, the total number of matches declines, because fewer database proteins remain that meet minimum BLAST requirements. As total number of Enterobacterial matches declines, matches to other classes of bacteria increase because they are the best remaining alternative. The maximum LPI value (LPI_max_), which is assigned to the lineage with the greatest number of matches, becomes progressively lower as the self-definition is expanded. The total number of matches having this LPI_max _value also declines, and the lineage associated with the LPI_max _becomes phylogenetically more distant from the original test genome. The histograms in Figure 3, grouped into bins of 0.02 units, show how the overall distribution of LPI scores changes from high to low as the number of closely related database taxa are depleted by broader self-definition terms. In this respect, using a coarser set of self-exclusion terms for an abundantly represented organism mimics the distribution of organisms that are more sparsely represented in the database.

Table 4 illustrates how changing self-definition keywords affects predictions of horizontal transfer for some individual protein examples. The first two rows in Table 4 contain sequences that are highly conserved among all strains of *E. coli*, as well as many closely related species. Matches to protein AAC75738 have lower e-values than matches to AAC74994 simply because AAC75738 is a much shorter protein (61 versus 495 amino acids). In these two rows, self-definition keywords do not affect LPI scores, which remain at maximum for both keyword sets.

LPI scores are also unchanged by self-definition keywords for the query sequences shown in rows 3 and 4, but for a different reason. Both of these sequences appear likely to have been recently acquired by *E. coli *strain K12, since its divergence from other *E. coli *strains. The closest database alignments to protein AAC75802 are with two species of delta-Proteobacteria, *Geobacter sulfurreducens *and *Desulfuromonas acetoxiadans *(not shown). This protein does not align well with any other strain of *E. coli*, nor with any other Enterobacterial genomes. Gene loss from such a large number of species seems unlikely as an alternative explanation to horizontal transfer.

Protein AAC75097 also appears to have been recently acquired by strain K12. Its origin is unclear; it aligns closely not only with a protein from *Psychromonas ingrahamii*, found in polar ice, but also with multiple examples among gamma-proteobacteria (*Actinobacillus succinogenes *and *Mannheimia succiniciproducens*), as well as epsilon-proteobacteria (*Campylobacter jejuni*) and eubacteria (several *Lactobacillus *and *Streptococcus *species). These organisms or their relatives could all potentially be found in human or bovine gut microflora, providing ample opportunity for gene exchange with both *E. coli *and each other. Differences in nucleotide composition between the proteins in rows 3 and 4 and the consensus for *E. coli *strain K12 (approximately 50% GC) also support recent lateral acquisition. Genomes from eubacteria in the Bacillus and Lactobacillus groups typically have a mean GC content around 35%.

The fifth row in Table 4 illustrates an example of likely horizontal gene transfer that occurred less recently. Using the narrowest set of self-definition keywords, protein AAC76015 has an LPI score of 0.993, equal to the LPI_max_, but the score drops substantially when the self-definition is expanded to include all species in the genus *Escherichia*. Closest alignments to this protein are found in multiple species of gamma-proteobacteria from the Pseudomonas lineage, but not in any other Enterobacteria besides *E. coli *strains K12, 536, UTI89, and F11. The atypically high GC percentage of this *E. coli *sequence is also consistent with transfer from members of genus *Pseudomonas*, whose genomes typically have mean GC contents of 60% or higher.

**Figure 4:**
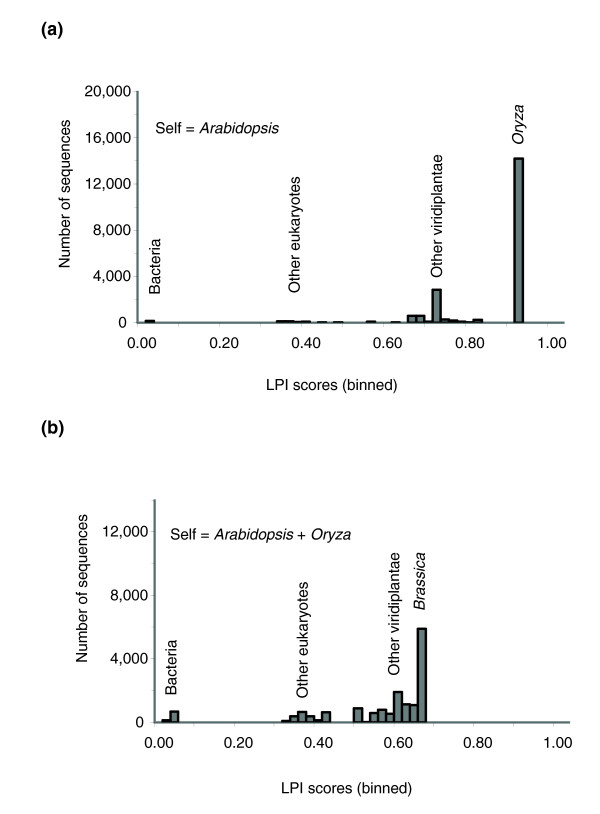
Effect of expanding *A. thaliana *self definition terms on LPI score distribution histograms. Filter threshold setting was 10%. **(a) **Self = *Arabidopsis*. **(b) **Self = *Arabidopsis *+ *Oryza*.

Table 5 illustrates a similar keyword expansion experiment performed with *Arabidopsis thaliana*. Adding *Oryza *to the self-definition list increases the number of bacterial matches from 162 to 812. Of these 812 matches, 336 are to cyanobacterial species, perhaps reflecting historical migration of chloroplast sequences derived from bacterial endosymbionts to the plant nucleus prior to the divergence of *Arabidopsis *and *Oryza*. The histograms in Figure 4 show how expanding the self definition not only lowers the top LPI scores, but also clarifies the separation of matches into three distinct groups, representing viridiplantae (scores 0.5 to 0.7), metazoan, fungal, and apicomplexan eukaryotes (scores 0.3 to 0.4), and bacteria (scores below 0.03).

One limitation to the technique of expanding self-definition terms is that it also reduces the total number of non-self BLAST matches. More than 90% of the original *E. coli *query sequences still have database matches above the BLAST initial screening criteria after excluding the three closest genera, but adding just a single genus to the *Arabidopsis *self-definition eliminated 20% of the original matches. For phylogenetic groups with less extensive database representation, exclusion of too many related groups may reduce the number of matches to a point where it is too low to reasonably represent the test genome.

### LPI score significance

The DarkHorse algorithm does not provide explicit criteria for classifying sequences as horizontally transferred or not; rather it ranks all candidates within a genome relative to each other. Selecting a single absolute value as a universal cutoff between positive and negative candidates for horizontal transfer neither makes biological sense, nor can it be supported computationally in the absence of unambiguous, known, and generally accepted positive and negative examples. Score distributions vary widely according to the evolutionary history of a test organism, the definition of 'self' chosen, and the number of closely related sequences in the database that lie outside that definition of self for a particular query.

**Figure 5:**
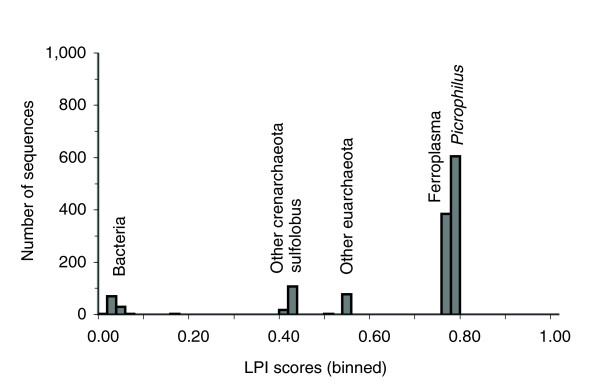
LPI score distribution histogram for *T. acidophilum*. Filter threshold setting was zero.

**Figure 6:**
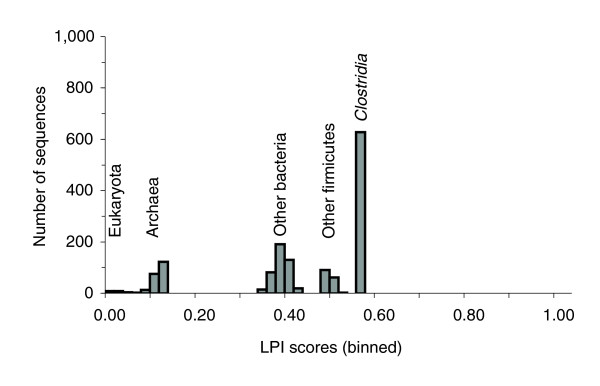
LPI score distribution histogram for *T. maritima*. Filter threshold setting was zero.

**Figure 7:**
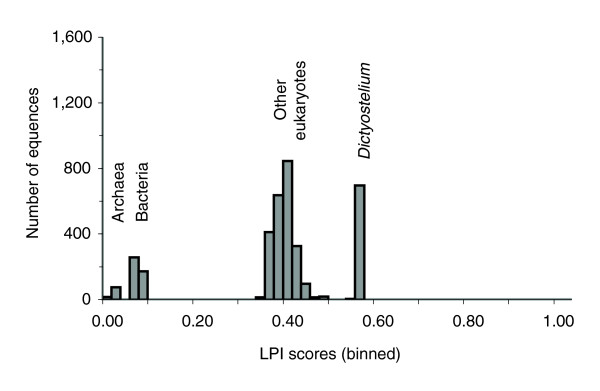
LPI score distribution histogram for *E. histolytica*. Filter threshold setting was zero.

Despite the difficulty of defining exact classification boundaries, some solid general principles can be applied to interpreting LPI score distributions, as illustrated by histograms of binned data in Figures 3 to 7. Query protein sequences with the highest LPI scores (LPI_max_) can be eliminated from consideration as horizontal transfer candidates with a high degree of confidence, because they are matched with proteins from lineages most closely related to the query organism. By definition, LPI scores must fall between zero and one. Within these limits, LPI_max _values cover a fairly broad range, with lower scores characteristic of organisms with few close relatives in the database, or with self-definition settings that have intentionally filtered out the closest relative sequences. Query protein sequences with intermediate LPI scores may or may not have been horizontally transferred, and will require analysis by independent methods to classify definitively. The number of query proteins with intermediate scores typically decreases as more closely related genomes are added to the underlying database. Scores at the lowest end of the LPI score distribution represent the best candidates for horizontal transfer, because their closest database matches belong to lineages that are most distantly related to the query organism. In the most extreme cases, if the closest match falls in a different kingdom, these sequences can have scores of 0.1 or lower.

### Bacterial and Archaeal examples

Two microbial organisms previously demonstrated by multiple bioinformatics methods to have high rates of horizontal gene transfer were re-analyzed for comparison using the DarkHorse algorithm. Euryarchaeotal species *Thermoplasma acidophilum *has been suggested to have experienced lateral gene exchange specifically with *Sulfolobus solfataricus*, a distantly related crenarchaeote that lives in the same ecological niche [39]. The hyperthermophilic bacterium *Thermotoga maritima *is believed to have undergone particularly high rates of horizontal gene exchange with archaeal species sharing its extreme habitat [40-42]. Each of these genomes was analyzed using its genus name as a self-exclusion term, and filter threshold cutoff values ranging from 0% to 40%.

The 1,494 predicted protein sequences of *T. acidophilum *had numerous best matches to distantly related organisms, including both *Sulfolobus*, as expected, and a variety of bacterial species (Table 6, Figure 5; raw data in Additional data file 2). Using a filter threshold of zero, the LPI score for the *Sulfolobus *lineage was 0.42, substantially below the *Picrophilus *and *Ferroplasma *lineages, with LPI scores of 0.76 to 0.79. The number of query proteins with best matches to *Sulfolobus *proteins was 106, consistent with a previous study that found 93 laterally transferred proteins agreed upon by three different prediction methods, with an additional 90 agreed upon by two out of the three methods [34]. In addition, DarkHorse analysis identified 97 query sequences most closely matched to bacterial proteins that were not examined in previous studies. These matches included species like *Thermotoga maritima*, which may themselves have acquired archaeal sequences from a *Thermoplasma *relative. This multi-level data complexity undoubtedly contributes to the inconsistency of horizontal transfer predictions from different bioinformatic methods.

Table 7 and Figure 6 summarize LPI score distributions for *Thermotoga maritima *(raw data provided in Additional data file 3). Database matches scoring above the minimum BLAST criteria were found for 1,440 (78%) of 1,846 predicted proteins in the *Thermotoga *genome. With a cutoff filter value of 0, the majority of matches, 617, were to bacteria of the Firmicutes/Clostridia lineage, generating LPI scores of 0.54 to 0.55 for these lineages. An LPI_max _value of 0.55 is much lower than that observed for many other microbial genomes, reflecting the absence of a truly close relative in the source database. The most abundant genus in the Clostridia group was *Thermoanaerobacter*, but this genus had only 265 matches. Other bacterial species from the Firmicutes lineage had LPI scores of 0.46 to 0.50, and more distant bacterial lineages had LPI scores between 0.33 and 0.41. At the lowest end of the score distribution were 208 matches to archaeal sequences, with LPI values of 0.1 or less. These archaeal matches represented 11.3% of the *Thermotoga *genome, consistent with previous reports suggesting that between 11% and 24% of proteins in this species have been laterally acquired [1,41]. The wide variability in literature predictions for numbers of horizontally transferred genes reflects the difficulty of assigning definitive classifications by any single bioinformatic method. However, LPI score distributions have captured and quantified the scarcity of orthologous sequences from closely related species in the source database, an important factor contributing to this discrepancy.

### Eukaryotic examples

The parasitic amoeba *Entamoeba histolytica *is believed to have lost its mitochondria and many enzymes associated with aerobic metabolism as an adaptation to its parasitic lifestyle and anaerobic habitat in the human gut. At the same time, this organism appears to have gained a set of enzymes not found in other eukaryotes, supporting anaerobic fermentation pathways. These enzymes may have been obtained by lateral gene transfer from phagocytized bacterial prey. In support of this hypothesis, a previous study has identified 96 genes considered most likely to have been laterally acquired, using a combination of automated and manual phylogenetic methods [43].

To compare DarkHorse predictions with those obtained by other methods, the *E. histolytica *genome was analyzed using the genus name as a self-definition, and filter threshold settings of 0% to 40%. Out of 9,775 predicted protein sequences, only 3,573 (37%) had matches above the minimum BLAST criteria, reflecting the scarcity of database sequence relatives. The maximum number of best matches to a single query rose abruptly from 33 to 497 when raising the threshold filter setting from 0% to 2%. These results suggest that database coverage for this organism is so sparse that filter settings higher than zero, shown in Table 8, are probably too lenient.

The LPI score distribution for *E. histolytica *is divided into several distinct phylogenetic clusters (Figure 7; raw data in Additional data file 4). The low LPI_max _value of 0.56, associated with 694 matches to genus *Dictyostelium*, confirms the scarcity of related species in the database. Best matches with LPI scores between 0.3 and 0.5 were associated with a wide diversity of other eukaryotic organisms, including plants, animals, and fungi as well as protozoa. The bacterial cluster of best matches had LPI scores between 0.04 and 0.07, and archaeal best matches had scores below 0.02. Previous work did not distinguish between archaeal and bacterial matches in *E. histolytica*, but grouped them all together among the 96 predicted lateral transfer candidates. Finding the archaeal sequence matches is particularly interesting, because they represent potential evidence supporting the theory of archaeal contributions to virulence in bacterial human pathogens [10].

Using a zero filter threshold cutoff, DarkHorse found non-eukaryotic best matches for 86 of the 96 *E. histolytica *genes previously identified as lateral transfer candidates. Of the ten differences, four were due to revisions in *E. histolytica *gene models - the older predicted *Entamoeba *sequences are no longer present in the current GenBank version of the genome. One disagreement occurred because the bacterial match proposed by Loftus *et al*. did not pass the initial DarkHorse BLAST pre-screening criteria for orthology, with an alignment length covering less than 60% of the query sequence [43]. One of the remaining five differences was found by DarkHorse to have a best match in *Mastigamoeba balamuthi*, and the remaining four to proteins in *Dictyostelium discoideum*. These are both amoeboid species representing close database relatives of *E. histolytica*. If these five *E. histolytica *sequences were laterally acquired, it must have been prior to evolutionary divergence from other eukaryotic ameboid species. It is possible that the *Dictyostelium *and *Mastigamoeba *sequence matches missed by previous analysis were not yet available at the time the work was done, therefore representing false positives. If so, this highlights the importance of re-analyzing phylogenetic data as new sequences for relatives of the query organism become available.

The most abundant bacterial and archaeal matches in the *E. histolytica *genome were to species known to inhabit the human digestive tract, including oral pathogen *Tannerella forsythensis *(45 matches), gut symbiont *Bacteroides thetaiotaomicron *(21 matches), and archaea from the genus *Methanosarcina *(40 matches). All 45 *T. forsythensis *matches point to a single bacterial cell surface-associated protein, BspA, previously shown to mediate dose-dependent binding to the human extracellular matrix components fibronectin and fibrinogen [44]. Sixteen best matches in *Methanosarcina *point to archaeal relatives of this same protein. Interestingly, there were no DarkHorse best matches to *T. forsythensis *or BspA in the genome of *Dictyostelium discoideum*, and only five matches to *B. thetaiotaomicron *and three to *Methanosarcina*.

The true biological relationships involved in *E. histolytica *gene evolution are quite complex, probably including multiple horizontal transfer events between eukaryotes, archaea, and bacteria that may themselves contain previously acquired archaeal sequences. Using a filter threshold setting of zero, DarkHorse identified an additional 60 archaeal and 350 bacterial best matches that were not described in the original *E. histolytica *genome paper. The most likely reason for this discrepancy is sub-optimal sensitivity of Pyphy [33], the automated phylogenetic tree building software used by Loftus *et al*., when dealing with complex data sets [43]. The Pyphy tree-building parameters were originally designed to find simple paralogous sequence relationships between closely related clades. Lower than expected Pyphy sensitivity has been described by other authors attempting to use it for horizontal gene transfer analysis across wide phylogenetic distances [34].

## Discussion

The algorithm presented here combines sequence alignment, database mining, statistical, and linguistic analysis tools in a single unified application. It compensates for differences in protein conservation by using BLAST scores in a relative, rather than an absolute context, with uniquely determined criteria for each genomic protein being tested. BLAST scores are used to define a set of candidate matches for each test protein, which are then ranked using a second, independent method, based on lineage frequency of matches over the entire genome. The power of the algorithm resides in its ability to integrate sequence alignments for individual proteins with phylogenetic statistics for an entire genome into a single quantitative metric, the LPI score, in a computationally efficient manner.

Sensitivity can be adjusted by restricting or broadening a filter threshold setting for candidate matches to compensate for differences in database representation of closely related organisms or for taxon-specific variability in mutation rates, which can mask horizontal transfer events or cause false positives. The method can be tuned to detect broader or narrower phylogenetic distance, as well as earlier versus more recent historical events, by expanding or contracting initial terms used for definition of 'self'. This flexibility facilitates adaptation of the program to a variety of different research goals, asking different kinds of questions.

The DarkHorse algorithm incorporates consensus knowledge of lineage relationships previously established from other, independent sources. The price for incorporating this information is a crucial dependence on the availability, quality, and timely updates of underlying sequence and taxonomy databases. All phylogenetic methods share this same dependence, although it is often unrecognized. One advantage of the DarkHorse method is that it combines the statistical power of thousands of database comparisons with a weighting scheme that maximizes the contribution of the broadest, most well-established classifications, and minimizes potential artifacts arising from fine-grained details that may be controversial or incorrect. This strategy provides a robust calculation of global lineage probabilities over an organism's entire genome, even in the presence of minor database errors for individual sequences or species. It can also be useful in identifying database mistakes that need to be corrected, as shown by the vector contamination examples in Table 2.

Some phylogenetic groups that undoubtedly participate in horizontal transfer, especially bacteriophages and other viruses, are not yet associated with sufficient taxonomy information to allow lineage analysis. False positive predictions of horizontal transfer may occur in cases of insufficient database coverage, where related species that contain orthologous proteins exist in real life, but are not included in the database at the time of analysis. Loss of individual genes in closely related species is also a potential problem, although mitigated by the thoroughness of the DarkHorse search algorithm, which incorporates data from all entries for all taxa in the database for every protein query.

By design, the LPI ranking system is less sensitive to transfer between closely related organisms than more distant ones, and does not attempt to establish directionality of lateral transfer events. Ranking of horizontal transfer candidates in a genome is relative; no absolute cutoff thresholds for classification can be computationally justified in the absence of unambiguous, known, and generally accepted positive and negative examples. For these reasons, subsequent validation of horizontal transfer candidates by alternative methods is essential to ensure accuracy of final determinations.

The biology of lateral transfer between genomes is emerging as a highly complex process, with little or no opportunity to perform experimental validation of bioinformatic predictions. Addressing this complexity effectively requires the power of combining multiple analytical approaches. The toolbox of every researcher needs to include reliable methods for constructing phylogenetic trees at widely varying distances, identifying and comparing genomic signatures, determining gene location synteny between closely related species, and defining the environmental conditions and lifestyle opportunities that might allow lateral transfer to occur between individual organisms.

The DarkHorse algorithm makes some unique contributions to the researcher's toolbox that are not provided by other techniques. LPI score distributions capture an important, potentially confounding piece of information that is neither collected nor recognized by other analytical methods, namely quantifying the density of current database coverage for potential relative organisms as a source of protein orthologs. The exceptionally rapid processing, screening and ranking of very large phylogenetic data sets in an automated manner makes it practical to analyze eukaryotic, as well as microbial genomes, and to perform repeated analyses as external databases are updated. Output from the program can then be used to select and prioritize candidates for follow-up with more detailed, sophisticated methods that would be too time consuming to apply to whole genomes on an ongoing, repeated basis. Finally, the DarkHorse program provides an exhaustive search function that can be used to identify orthologs from other species that may have been omitted or unknown at the time of previous analyses. This application permits quality assurance testing to be performed retrospectively on previous studies using any and all other predictive methods to ensure that their conclusions still remain valid after the expansion of our knowledge by the addition of new sequence data.

## Materials and methods

### Genomes and databases

Predicted protein sequences for test genomes were downloaded from the NCBI GenBank genome website [45], with the exception of *D. discoideum*, which was downloaded from dictyBase [46] and *A. thaliana*, which was downloaded from the TIGR *Arabidopsis thaliana *Database [47]. GenBank protein sequences and their associated species information (the nr and taxdb databases) were obtained from the NCBI BLAST database [48]. NCBI taxonomy database tables were downloaded from the NCBI taxonomy database [49].

### Software

BLAST searches were performed using either the DeCypher Tera-BLAST™ (TimeLogic, Inc. Carlsbad, California, USA) or NCBI BLAST program. Species names associated with BLAST matches were retrieved using the fastacmd module of the NCBI BLAST program. NCBI taxonomy data tables were entered into a local installation of the MySQL relational database program using a custom perl script. Lineages were retrieved for individual species using a recursive perl script that traversed the taxonomy tree through the database to its root level, producing output similar to lineage information available through the NCBI taxonomy website. Software to perform lineage probability index (LPI) calculations has been implemented as a perl-scripted pipeline for the UNIX operating system, with links to local hardware-accelerated BLAST search software and local MySQL databases. A more generalized integrated software interface is under development.

### Computing resources

The rate-limiting step for the current procedure is a BLAST search of all predicted proteins from a test genome against the GenBank nr protein sequence database, collecting as many as 500 hits per query sequence. This step was performed using a DeCypher hardware-accelerated Tera-BLAST™ system, but could also be done using a multiprocessor cluster, or any other hardware configuration capable of acceptable BLAST performance with large data sets. With the DeCypher system, typical BLAST search times for a test set of 5,000 predicted proteins against the GenBank nr database (currently 3.5 million sequences) were around 30 minutes. The remainder of the analysis can typically be completed in 10 to 60 minutes, depending on genome size, using a single CPU on a Sun V440 Unix workstation (1.3 GHz, 16 GB RAM). This stage requires no special hardware; most of the time is spent on SQL query retrieval from the MySQL relational database.

### Calculation of lineage probabilities

The main steps of the overall algorithm are summarized in Figure 1, and described in the Results sections called 'Algorithm overview' and 'Selection and ranking of candidate match sets'. The steps used to calculate normalized, weighted, lineage probabilities are the same for both preliminary guide probabilities and final LPI scores. These steps are described in detail below, using the contents of Table 9 as an example.

#### Step 1

Determine the average hierarchical position of each lineage term. The numbers start at one, ordered from left to right, so that the most general term has the lowest number, and the most specific term has the highest number. In the Table 9 examples, the terms 'Bacteria', 'Eukaryota' and 'Viruses' are assigned to position one, 'Actinobacteria', 'Cyanobacteria', 'Dictyosteliida', 'Myxogastromycetidae' and 'Caudovirales' are assigned to position two, and so forth.

#### Step 2

Count the total number of entries for each hierarchical position in the whole set. Positions 1 to 3 in this example each contain six entries, because all six sequences on the list have at least three terms. Position 4 contains only three entries (from sequences number 2, 3 and 5), and position 5 contains only one entry ('Nostoc', from sequence 2).

#### Step 3

Determine the frequency (number of occurrences) for each individual term in the whole set. In this example, the term 'Bacteria' has a frequency of 3, 'Eukaryota' and 'Cyanobacteria' each has a frequency of 2, and all of the other terms have a frequency of 1.

#### Step 4

Calculate raw probability by dividing term frequency by the total number of entries for the term's hierarchical position. The maximum possible probability for each lineage term is, therefore, 1.0. In this example, the raw probability for the term 'Bacteria' is 3/6 (0.5). 'Eukaryota' and 'Cyanobacteria' both have a raw probability of 2/6 (0.33), and 'Viruses' has a raw probability of 1/6 (0.17). However, the term 'Nostocaceae' has a raw probability of 1/3 (0.33), because there are only three possible terms at position 4.

#### Step 5

Divide each term's raw probability by its hierarchical position to give a weighted probability value. This gives the highest weight to the most general terms. In this example, the term 'Eukaryota' receives a weighted probability of 0.33/1 (0.33), but the weighted probability of 'Cyanobacteria' is only 0.33/2 (0.16), because it is in the second hierarchical position.

#### Step 6

For each unique lineage, add together the weighted probabilities of all component terms to calculate a composite probability. The composite probabilities of each of the example lineages are as follows:

0.50 + 0.08 + 0.06 = 0.64

0.50 + 0.16 + 0.06 + 0.08 + 0.20 = 1.00

0.50 + 0.16 + 0.06 + 0.08 = 0.80

0.33 + 0.08 + 0.06 = 0.47

0.33 + 0.08 + 0.06 + 0.08 = 0.55

0.22 + 0.08 + 0.06 = 0.36

#### Step 7

To account for lineages that have different numbers of terms, divide each composite probability by a length normalization factor, equal to the sum of reciprocal values for the number of composite terms it contains. As an example, for lineages with three terms, the length normalization factor is 1/1 + 1/2 + 1/3 = 1.83, so the final LPI score for lineage number 1 will be 0.64/1.83 = 0.35. For lineages with five terms, the length normalization factor is 1/1 + 1/2 + 1/3 + 1/4 + 1/5 = 2.28, so the final LPI score for lineage number 2 is 1.00/2.28 = 0.44.

For a small minority of data points, species and/or lineage information may be absent from the database. These protein matches are excluded from lineage probability calculations, since they are not informative. In practice, these sequences will often be annotated as 'uncultured bacterium' or 'cloning vector'. These entries are flagged and saved to a log file, allowing the user to decide whether the taxonomy database needs to be updated to a newer version, or the entries are insignificant and can be added to the automatic exclusion list. Final output is formatted as a tab-delimited file containing the following information: query id, total number of BLAST hits, number of non-self BLAST hits, number of candidate matches, initial tophit id, corrected best hit id, LPI score, percent identity of the BLAST match, query sequence length, alignment length, alignment coverage, e-value, bit score, taxonomy id, species, lineage, query annotation, and best match annotation.

### User-adjustable parameters

Initial BLAST screening parameters against the sequence source database were chosen broadly, using an e-value cutoff of 1e-05 or better, with at least 60% of query length covered by the BLAST alignment. These parameters may be adjusted if desired by the user; they serve merely as a pre-filter to remove matches of obvious low quality. The maximum number of saved alignments per query was 500 sequences for the analyses presented here, but this number may need to be increased for very large genomes.

## Additional data files

The following additional data are available with the online version of this paper. Additional data file 1 contains tab-delimited raw output from DarkHorse analysis of *E. coli *strain K12, with a filter threshold setting of 10% and self definition set as '*Escherichia*'. Additional data file 2 contains tab-delimited raw output for *Thermoplasma acidophilum*, with a filter threshold setting of zero and self definition set as '*Thermoplasma*'. Additional data file 3 contains tab-delimited raw output for *Thermotoga maritima*, with a filter threshold setting of zero and self definition set as '*Thermotoga*'. Additional data file 4 contains tab-delimited raw output for *Entamoeba histolytica*, with a filter threshold setting of zero and self definition set as '*Entamoeba*'.

## Supplementary Material

Additional data file 1 The filter threshold setting was 10% and self definition was set as '*Escherichia*'. Click here for file

Additional data file 2 The filter threshold setting was zero and self definition was set as '*Thermoplasma*'. Click here for file

Additional data file 3 The filter threshold setting was zero and self definition was set as '*Thermotoga*'. Click here for file

Additional data file 4 The filter threshold setting was zero and self definition was set as '*Entamoeba*'. Click here for file

## Figures and Tables

**Table 1:** Effect of filter threshold setting on best match lineages for *E. coli*

	Filter threshold setting									
	
	0%	2%	5%	10%	20%	30%	40%	60%	80%	100%
Enterobacteria	4,000	4,034	4,052	4,063	4,064	4,078	4,092	4,105	4,112	4,112
Other bacteria	132	112	103	96	85	74	76	64	58	58
Phage	27	24	18	14	12	11	7	6	6	6
Eukaryotes	8	6	6	6	4	4	4	4	3	3
Archaea	0	0	0	0	0	0	0	0	0	0

Total matches	4,167	4,176	4,179	4,179	4,165	4,167	4,179	4,179	4,179	4,179

As discussed in the text, a zero percent filter threshold setting retains only candidates with bit scores equal to the top non-self blast match. A setting of 100% retains all matches as candidates for subsequent LPI calculations. Some columns have slightly lower total numbers due to matches with uncultured organisms, which contain no lineage information but were not filtered out in this experiment.

**Table 2:** Effect of filter threshold setting and LPI score ranking on eukaryotic BLAST matches to *E. coli*

Filter threshold	Query id	Match id	LPI	Percent identity	Query length	Align length	e-value	Bit score	Match species	Query annotation	Match annotation
**0.0**	**AAC74689**	**CAC43289**	**0.009**	**99**	**603**	**603**	**0**	**1261**	***Arabidopsis thaliana***	**Beta-glucuronidase**	**Beta-glucuronidase**
*0.02*	*AAC74689*	*ZP_00698534*	*0.981*	*99*	*603*	*603*	*0*	*1255*	*Shigella boydii*		*Beta-galactosidase/beta-glucuronidase*

**0.0**	**AAC76624**	**AAM52982**	**0.009**	**99**	**382**	**382**	**0**	**741**	***Dunaliella bardawil***	**Mannitol-1-phosphate dehydrogenase**	**Mannitol-1-phosphate dehydrogenase**
*0.02*	*AAC76624*	*AAN45081.2*	*0.981*	*98*	*382*	*382*	*0*	*738*	*Shigella flexneri*		*Mannitol-1-phosphate dehydrogenase*

**0.0**	**AAC73440**	**AAU04862**	**0.001**	**96**	**427**	**425**	**0**	**830**	***Tamarix chinensis***	**Cytosine deaminase**	**Cytosine deaminase**
*0.2*	*AAC73440*	*AAV79026*	*0.925*	*81*	*427*	*420*	*0*	*706*	*Salmonella enterica*		*Cytosine deaminase*

**0.0**	**AAC73353**	**AAA35359**	**0.088**	**78**	**155**	**99**	**7.0E-42**	**171**	***Cercopithecus aethiops***	**CP4-6 prophage**	**None**
*0.2*	*AAC73353*	*ZP_00825492*	*0.924*	*48*	*155*	*145*	*1.0E-36*	*153*	*Yersinia mollaretii*		*Hypothetical protein*

**0.0**	**AAC75891**	**gi|2143952**	**0.108**	**85**	**458**	**441**	**0**	**719**	***Rattus norvegicus***	**Predicted transcriptional regulator**	**Hepatic glutathione transporter**
*0.8*	*AAC75891*	*AAD12579*	*0.927*	*28*	*458*	*403*	*1.0E-38*	*164*	*Salmonella typhimurium*		*HilA*

**0.0**	**AAC73796**	**BAB33410**	**0.029**	**100**	**108**	**108**	**1.0E-54**	**213**	***Pisum sativum***	**Predicted inner membrane protein**	**Putative senescence-associated protein**

**0.0**	**AAC74583**	**BAE25662**	**0.104**	**92**	**1325**	**895**	**0**	**1614**	***Musmusculus***	**Predicted lipoprotein**	**none**

**0.0**	**ABD18679**	**gi|1095170**	**0.108**	**93**	**234**	**179**	**3.0E-86**	**320**	***Rattus norvegicus***	**Predicted protein, amino terminal fragment (pseudogene)**	**Glutathione transporter**

Rows in bold type contain the top ranked match using a zero threshold setting. Rows in *italic *type show cases where using a higher filter setting revealed an alternative match, with a higher LPI score, to the same genomic query.

**Table 3:** Effect of self-definition keywords on best match lineages for *E. coli*

	Self-definition keywords			
	K12 83333 316407 562	*Escherichia*	*Escherichia* *Shigella*	*Escherichia* *Shigella* *Salmonella*

Enterobacteria	4,203	4,063	3,640	3,173
Other bacteria	34	96	346	632
Phage	1	14	55	80
Eukaryotes	0	6	12	18
Archaea	0	0	2	3

Total matches	4,243	4,179	4,055	3,906
LPI_max_	0.993	0.984	0.950	0.918
LPI_max _matches	4,110	3,855	3,220	2,570

LPI_max _lineage	Bacteria; Proteobacteria; Gamma-proteobacteria; Enterobacteriales; Enterobacteriaceae; *Escherichia*	Bacteria; Proteobacteria; Gamma-proteobacteria; Enterobacteriales; Enterobacteriaceae; *Shigella*	Bacteria; Proteobacteria; Gamma-proteobacteria; Enterobacteriales; Enterobacteriaceae; *Salmonella*	Bacteria; Proteobacteria; Gamma-proteobacteria; Enterobacteriales; Enterobacteriaceae; *Yersinia*

Filter threshold setting was 10%.

**Table 4:** Effect of self-definition keywords on LPI scores for individual protein examples from *E. coli *strain K12

			Self-definition keywords					
			
			K12 83333 316407 562			*Escherichia*		
			
Query ID	Query annotation	Query GC%	Match species	LPI	e-value	Match species	LPI	e-value
AAC74994	Cytoplasmic alpha-amylase	49	*Escherichia coli CFT073*	0.993	0	*Shigella dysenteriae*	0.984	0
AAC75738	Carbon source regulatory protein	49	*Escherichia coli O157:H7*	0.993	3e-26	*Shigella flexneri*	0.984	3e-25
AAC75802	Conserved hypothetical protein	43	*Geobacter sulfurreducens*	0.612	3e-138	*Geobacter sulfurreducens*	0.610	3e-138
AAC75097	UDP-galactopyranose mutase	35	*Psychromonas ingrahamii*	0.747	2e-149	*Psychromonas ingrahamii*	0.743	2e-149
AAC76015	Glycolate oxidase subunit, FAD-linked	56	*Escherichia coli 53638*	0.993	0	*Pseudomonas syringae*	0.745	0

**Table 5:** Effect of self-definition terms on best match lineages for *A. thaliana*

	Self-definition keywords		
	
	*Arabidopsis*	*Arabidopsis* *Oryza*	*Arabidopsis* *Oryza* *Brassica*
Viridiplantae	19,229	12,078	11,658
Other Eukaryotes	583	3,122	3,191
Bacteria	162	812	850
Archaea	3	12	13
Viruses	1	2	3

Total matches	19,978	16,026	15,715
LPI_max_	0.907	0.671	0.670
LPI_max _matches	14,215	2,437	2,960

LPI_max _lineage	Eukaryota; Viridiplantae; Streptophyta; Liliopsida; commelinids; Poales; Poaceae; Ehrhartoideae; Oryzeae; *Oryza*	Eukaryota; Viridiplantae; Streptophyta; rosids; Brassicales; Brassicaceae; *Brassica*	Eukaryota; Viridiplantae; Streptophyta; asterids; Solanales; Solanaceae; *Solanum*

Filter threshold setting was 10%.

**Table 6:** Effect of filter threshold on best match lineages for *T. acidophilum*

	Filter threshold setting					
	
	0%	2%	5%	10%	20%	40%
Picrophilus	604	658	760	852	919	976
Sulfolobus	106	104	81	76	50	40
Other Archaea	483	437	373	302	267	236
Bacteria	97	92	78	62	54	37
Eukaryotes	4	3	3	3	5	6

Total matches	1,294	1,294	1,295	1,295	1,295	1,295

As in Table 1 for *E. coli*, a zero percent filter threshold setting retains only candidates with bit scores equal to the top non-self blast match. A setting of 100% retains all matches as candidates for subsequent LPI calculations. Some columns have slightly lower total numbers due to matches with uncultured organisms, which contain no lineage information but were not filtered out in this experiment.

**Table 7:** Effect of filter threshold setting on best match lineages for *T. maritime*

	Filter threshold setting					
	
	0%	2%	5%	10%	20%	40%
Clostridia	627	695	799	917	1,064	1,170
Other Firmicutes	135	115	99	79	55	56
Non-Firmicutes bacteria	458	422	364	300	229	170
Archaea	208	197	172	139	89	46
Eukaryotes	12	11	7	6	5	1

Total matches	1,440	1,440	1,441	1,441	1,442	1,443

Some columns have slightly lower total numbers due to matches with uncultured organisms, which contain no lineage information but were not filtered out in this experiment.

**Table 8:** Effect of filter threshold setting on best match lineages for *E. histolytica*

	Filter threshold setting					
	
	0%	2%	5%	10%	20%	40%
Dictyostelium	694	831	1,096	1,485	1,901	2,083
Other Eukaryotes	2,353	2,236	2,011	1,682	1,347	1,267
Bacteria	433	431	413	377	308	213
Archaea	72	61	50	35	22	11

Total matches	3,552	3,559	3,570	3,579	3,578	3,574

Some columns have slightly lower total numbers due to matches with uncultured organisms, which contain no lineage information but were not filtered out in this experiment.

**Table 9:** Examples of NCBI taxonomy lineages

Species	Number of terms	Lineage
*Symbiobacterium thermophilum*	3	Bacteria;Actinobacteria;*Symbiobacterium*
*Nostoc punctiforme*	5	Bacteria;Cyanobacteria;Nostocales;Nostocaceae;*Nostoc*
*Trichodesmium erythraeum*	4	Bacteria;Cyanobacteria;Oscillatoriales;*Trichodesmium*
*Dictyostelium discoideum*	3	Eukaryota;Dictyosteliida;*Dictyostelium*
*Physarum polycephalum*	4	Eukaryota;Myxogastromycetidae;Physariida;*Physarum*
*Enterobacteria phage P1*	3	Viruses;Caudovirales;*Myoviridae*
